# Supplementary material for: Do latitudinal gradients exist in New Zealand stream invertebrate metacommunities?
Source: PeerJ. 2018 May 25;6:e4898. doi: 10.7717/peerj.4898 (PMC5971837; doi:10.7717/peerj.4898)
Supplement: Supplemental Information 1 — Results of forward selection on both spatial and environmental variables for the eight metacommunities. If a global model was significant, forward selection was performed. Forward-selected variables are given in the “Variables” column. Subset = subset of species (all, and strong and weak dispersers). Global model sig. = Global model significant or not (N: no; Y: yes). [file peerj-06-4898-s001.pdf]

Supporting Information

Do latitudinal gradients exist in New Zealand stream invertebrate metacommunities?

Jonathan D. Tonkin, Russell G. Death, Timo Muotka, Anna Astorga, and David A. Lytle

Appendix S1: Full results of forward selection for variance partitioning.

Table S1: Results of forward selection on both spatial and environmental variables for the eight metacommunities. If a global model was significant, forward selection was performed. Forward-selected variables are given in the "Variables" column. Subset = subset of species (All, and strong and weak dispersers). Global model sig. = Global model significant or not (N: no; Y: yes).

Subset	Region	Data	F	P	Model	Sig.	Variables
All	N	Environmental	1.02	0.50	Global	N	
All	N	Spatial	0.90	0.69	Global	N	
All	U	Environmental	1.46	0.03	Global	Y	
All	U	Environmental	2.57	0.00	Fwd. sel.		Temp, pH
All	U	Spatial	1.60	0.02	Global	Y	
All	U	Spatial	2.39	0.00	Fwd. sel.		PCNM1, PCNM3
All	E	Environmental	1.72	0.03	Global	Y	
All	E	Environmental	2.96	0.00	Fwd. sel.		OHCov, Elev, SI, Depth
All	E	Spatial	0.84	0.73	Global	N	
All	T	Environmental	1.19	0.28	Global	N	
All	T	Spatial	0.76	0.86	Global	N	
All	K	Environmental	1.66	0.02	Global	Y	
All	K	Environmental	2.25	0.00	Fwd. sel.		Cond, OHCov
All	K	Spatial	0.81	0.85	Global	N	
All	A	Environmental	1.80	0.02	Global	Y	
All	A	Environmental	2.64	0.03	Fwd. sel.		Temp
All	A	Spatial	1.97	0.00	Global	Y	
All	A	Spatial	3.29	0.00	Fwd. sel.		PCNM1, PCNM2, PCNM3
All	W	Environmental	2.08	0.03	Global	Y	
All	W	Environmental	4.55	0.00	Fwd. sel.		Cond, pH, Slope
All	W	Spatial	1.80	0.05	Global	Y	
All	W	Spatial	2.70	0.03	Fwd. sel.		PCNM9
All	F	Environmental	1.78	0.01	Global	Y	
All	F	Environmental	2.13	0.01	Fwd. sel.		Order
All	F	Spatial	1.33	0.12	Global	N	
Strong	N	Environmental	1.07	0.42	Global	N	
Strong	N	Spatial	0.96	0.58	Global	N	
Strong	U	Environmental	1.47	0.07	Global	N	
Strong	U	Spatial	1.71	0.02	Global	Y	
Strong	U	Spatial	2.64	0.00	Fwd. sel.		PCNM1, PCNM3
Strong	E	Environmental	2.07	0.03	Global	Y	
Strong	E	Environmental	3.83	0.00	Fwd. sel.		OHCov, Elev, SI
Strong	E	Spatial	0.81	0.74	Global	N	
Strong	T	Environmental	1.02	0.45	Global	N	
Strong	T	Spatial	0.66	0.92	Global	N	
Strong	K	Environmental	1.84	0.02	Global	Y	
Strong	K	Environmental	2.64	0.01	Fwd. sel.		Cond, Chla
Strong	K	Spatial	0.59	0.96	Global	N	
Strong	A	Environmental	2.18	0.02	Global	Y	
Strong	A	Environmental	3.20	0.04	Fwd. sel.		Temp
Strong	A	Spatial	2.27	0.01	Global	Y	
Strong	A	Spatial	4.48	0.00	Fwd. sel.		PCNM1, PCNM2
Strong	W	Environmental	1.82	0.09	Global	N	
Strong	W	Spatial	1.62	0.12	Global	N	
Strong	F	Environmental	1.78	0.08	Global	N	
Strong	F	Spatial	1.20	0.35	Global	N	
Weak	N	Environmental	1.16	0.28	Global	N	

Continued on next page

Subset	Region	Data	F	P	Model	Sig.	Variables
Weak	N	Spatial	0.82	0.82	Global	N	
Weak	U	Environmental	1.64	0.02	Global	Y	
Weak	U	Environmental	3.32	0.00	Fwd. sel.		Temp, pH
Weak	U	Spatial	1.63	0.02	Global	Y	
Weak	U	Spatial	2.50	0.00	Fwd. sel.		PCNM1, PCNM5, PCNM2
Weak	E	Environmental	1.37	0.18	Global	N	
Weak	E	Spatial	0.78	0.81	Global	N	
Weak	T	Environmental	1.71	0.04	Global	Y	
Weak	T	Environmental	2.57	0.00	Fwd. sel.		OHCov, Pfankuch_bottom, Chla, Depth
Weak	T	Spatial	0.82	0.77	Global	N	
Weak	K	Environmental	1.54	0.05	Global	Y	
Weak	K	Environmental	2.20	0.02	Fwd. sel.		Cond
Weak	K	Spatial	1.31	0.10	Global	N	
Weak	A	Environmental	1.05	0.42	Global	N	
Weak	A	Spatial	1.60	0.02	Global	Y	
Weak	A	Spatial	2.49	0.00	Fwd. sel.		PCNM1, PCNM5, PCNM2
Weak	W	Environmental	1.17	0.24	Global	N	
Weak	W	Spatial	1.44	0.03	Global	Y	
Weak	W	Spatial	1.67	0.02	Fwd. sel.		PCNM6
Weak	F	Environmental	1.71	0.02	Global	Y	
Weak	F	Environmental	2.13	0.02	Fwd. sel.		Order
Weak	F	Spatial	1.44	0.07	Global	N	
